# Modulation of soleus stretch reflexes during walking in people with chronic incomplete spinal cord injury

**DOI:** 10.1007/s00221-019-05603-1

**Published:** 2019-07-15

**Authors:** Aiko K. Thompson, N. Mrachacz-Kersting, T. Sinkjær, J. B. Andersen

**Affiliations:** 1 College of Health Professions, Medical University of South Carolina, 77 President Street, MSC700, Charleston, SC 29425 USA; 2 Center for Sensory-Motor Interaction, Aalborg University, 9220 Aalborg, Denmark; 3 Lundbeck Foundation, Scherfigsvej 7, 2100 Copenhagen, Denmark

**Keywords:** Spasticity, Hyperreflexia, Chronic incomplete spinal cord injury, Locomotion

## Abstract

In people with spasticity due to chronic incomplete spinal cord injury (SCI), it has been presumed that the abnormal stretch reflex activity impairs gait. However, locomotor stretch reflexes across all phases of walking have not been investigated in people with SCI. Thus, to understand modulation of stretch reflex excitability during spastic gait, we investigated soleus stretch reflexes across the entire gait cycle in nine neurologically normal participants and nine participants with spasticity due to chronic incomplete SCI (2.5–11 year post-injury). While the participant walked on the treadmill at his/her preferred speed, unexpected ankle dorsiflexion perturbations (6° at 250°/s) were imposed every 4–6 steps. The soleus *H*-reflex was also examined. In participants without SCI, spinal short-latency “*M*1”, spinal medium latency “*M*2”, and long-latency “*M*3” were clearly modulated throughout the step cycle; the responses were largest in the mid-stance and almost completely suppressed during the stance-swing transition and swing phases. In participants with SCI, *M*1 and *M*2 were abnormally large in the mid–late-swing phase, while *M*3 modulation was similar to that in participants without SCI. The *H*-reflex was also large in the mid–late-swing phase. Elicitation of *H*-reflex and stretch reflexes in the late swing often triggered clonus and affected the soleus activity in the following stance. In individuals without SCI, moderate positive correlation was found between *H*-reflex and stretch reflex sizes across the step cycle, whereas in participants with SCI, such correlation was weak to non-existing, suggesting that *H*-reflex investigation would not substitute for stretch reflex investigation in individuals after SCI.

## Introduction

Spinal reflexes are often altered after spinal cord injury (SCI) that disrupts the activity of supraspinal and propriospinal pathways (Yang et al. 1991; Stein et al. 1993; Hiersemenzel et al. 2000; Crone et al. 2003; Thompson et al. 2009b). Since the activity of spinal reflex pathways is task- and phase-dependently modulated in functionally relevant ways in normal motor control (Capaday and Stein 1986, 1987a; Stein and Capaday 1988; Duysens et al. 1990; Yang and Stein 1990; Zehr et al. 1997, 1998b; Zehr and Stein 1999b; Duysens et al. 2004) and different spinal pathways contribute to the generation of normal muscle activity during locomotion (Sinkjaer et al. 1996a; Grey et al. 2002, 2007; Mazzaro et al. 2005, 2006), the natural presumption has been that spinal reflex abnormalities contribute to impaired movement control (Dietz and Sinkjaer 2007; Nielsen et al. 2007). In fact, reflex abnormalities in the soleus of people after SCI have been described in multiple studies: absent or diminished task-dependent modulation of the *H*-reflex (Boorman et al. 1996; Thompson et al. 2009b); absence of Ib inhibition (Morita et al. 2006); abnormal reciprocal inhibition between the soleus and tibialis anterior (Ashby and Wiens 1989; Boorman et al. 1991, 1996; Crone et al. 2003; Thompson et al. 2009b); and increased recurrent inhibition (Shefner et al. 1992). Reflex abnormalities also exist in other muscles (Faist et al. 1999). Furthermore, observations of abnormal reflex behaviors (including motoneuronal and interneuronal activity) during static motor tasks or isolated joint motion (Hultborn 2003; Gorassini et al. 2004; Li et al. 2004; Hornby et al. 2006) have suggested several compelling potential mechanisms of movement disorders. Yet, findings from those studies do not provide direct evidence of reflex malfunctioning during dynamic motion. To better understand a reflex’s function during movement, the reflex must be examined during movement (Stein and Thompson 2006).

Spasticity, commonly characterized by a velocity-dependent increase in tonic stretch reflexes with exaggerated tendon jerks (Lance 1980), affects 65–78% of people after SCI (Maynard et al. 1990; Skold et al. 1999; Adams and Hicks 2005) and impairs their locomotion (this could be an over- or under-estimate of the spasticity occurrence, since different symptoms are likely reported as spasticity in clinical practice, including the hyperexcitability of tonic (e.g., hypertonia) and phasic (e.g., clonus) stretch reflexes of intrinsic and extrinsic origins (Decq 2003; Adams and Hicks 2005)). While several spinal and supraspinal pathways are thought to be involved in spastic movement disorders (Hultborn 2003; Dietz and Sinkjaer 2007; Nielsen et al. 2007; Burke et al. 2013; Li and Francisco 2015), how they contribute to gait impairments in SCI is not well understood. In people with SCI, the soleus stretch reflex excitability is often elevated in static postures or during hip rotation (Mirbagheri et al. 2001; Kawashima et al. 2006; Nakazawa et al. 2006), and clonus, hyperactivity, and abnormal modulation of the soleus *H*-reflex are frequently present (Corcos et al. 1986; Fung and Barbeau 1989, 1994; Yang et al. 1991; Stein et al. 1993; Hidler and Rymer 1999; Barbeau et al. 2002; Knikou et al. 2009a, b; Knikou 2013). Thus, to understand how the excitability of stretch reflex pathways is modulated during spastic gait, here, we investigated the soleus stretch reflexes across the entire gait cycle.

While the *H*-reflex is commonly viewed as the response generated through the same pathway as the short-latency stretch reflex and often used to investigate Ia excitation of homonymous motoneurons (Zehr 2002; Misiaszek 2003), the short-latency reflex (called *M*1) and *H*-reflex do not necessarily respond to modulatory input in the same way (Nielsen et al. 1994; Sinkjaer et al. 1996a; Morita et al. 1998). The *H*-reflex is an electrically elicited spinal reflex that arises mainly from synchronous axonal excitation of large Ia (and some Ib/II) afferents, whereas the stretch reflexes elicited by rapid joint rotation arise from less synchronous activation of Ia and II afferents and reflect γ-motoneuron mediated fusimotor control (Magladery et al. 1951; Henneman and Mendell 1980; McKeon and Burke 1983; Morita et al. 1998). Ib afferents arising from Golgi tendon organ also fire in response to muscle stretch, and excite motoneurons via interneurons (Lundberg et al. 1978; Jankowska and McCrea 1983; Schafer et al. 1999; Duysens et al. 2000; Jankowska and Edgley 2010; Vincent et al. 2017; Nichols 2018). In sum, while the afferent populations that elicit *H*-reflexes and stretch reflexes overlap, their compositions are not identical; furthermore, stretch reflex afferent excitation is less synchronous and is affected by fusimotor control of the muscle spindle. Thus, the study of both reflexes in parallel would provide further insight into their activation, modulation, and function during walking. To evaluate correlation and predictability of reflex excitability between the *H*-reflex and the stretch reflexes during dynamic motion, in this study, we examined across all phases of the gait cycle, the three components of soleus stretch reflexes: spinal short-latency “*M*1” (mainly Ia afferent origin), spinal medium latency “*M*2” (presumably mainly II afferent mediated), and long-latency “*M*3” (suggested to be transcortical or subcortical) responses (Corna et al. 1995; Sinkjaer et al. 1996b; Schieppati and Nardone 1997; Nardone and Schieppati 1998; Andersen and Sinkjaer 1999; Sinkjaer et al. 1999; Grey et al. 2001; Uysal et al. 2009; Af Klint et al. 2010; Uysal et al. 2011), together with the soleus *H*-reflex.

## Materials and methods

### Participants

Soleus *H*-reflex and stretch reflexes were examined in nine participants with no known neurological conditions (seven men and two women; age range 30–68 years; mean ± SD age 46.3 ± 11.9 years) and nine ambulatory participants with spasticity due to chronic incomplete SCI (eight men and one woman; age range 31–68 years; mean ± SD age 52.1 ± 11.3 years; 2.5–11 year post-injury, Table 1). The impairments of these participants fell into the American Spinal Injury Association Impairment Scale (AIS) D (i.e., motor incomplete). All participants gave informed consent for the study, which was reviewed and approved by the Institutional Review Board of Helen Hayes Hospital and the Medical University of South Carolina.

**Table 1:** Profiles of participants with chronic incomplete spinal cord injury (SCI)

Participant	Age	Sex	SCI cause	SCI level	AIS	Years post SCI	Baclofen	Treadmill speed (m/s)
1	68	M	NT	T5	D	11	Y	0.49
2	48	M	T	C1	D	2.5	Y	0.30
3	31	F	T	C7	D	9	Y	0.83
4	55	M	T	C5	D	5	N	0.54
5	45	M	T	C4	D	10	Y	0.54
6	67	M	T	C8	D	2	Y	0.49
7	48	M	T	T1	D	4	Y	1.00
8	52	M	T	C4	D	2.5	N	1.00
9	55	M	T	C6	D	10	N	0.40

Cause of spinal cord damage (*T* trauma, *NT* non-trauma)

*AIS* American Spinal Injury Association (ASIA) Impairment Scale

Inclusion criteria for participants with SCI were: (1) clinically stable (> 6 months after lesion); (2) medical clearance to participate; (3) ability to walk on the treadmill at 0.2 m/s or faster for at least 5 min at a time; (4) clinical sign of spasticity (i.e., Modified Ashworth Scale > 1) and/or occurrence of clonus more than once a day at the ankle at least unilaterally. Six of nine participants had been taking a stable dose of baclofen, with or without other anti-spastic medication (e.g., tizanidine and diazepam), for at least 6 months prior to the study. All of them had been medically stable with no change of medication for at least 3 months prior to the day of the experiment. Exclusion criteria were: (1) lower motor neuron injury; (2) known cardiac conditions; (3) medically unstable conditions; (4) cognitive impairment; and (5) extensive use of functional electrical stimulation (FES) foot-drop stimulator on a daily basis. In the participants who exhibited sign of spasticity bilaterally, the leg with more spasticity was investigated.

### Study overview

**Fig. 1:**
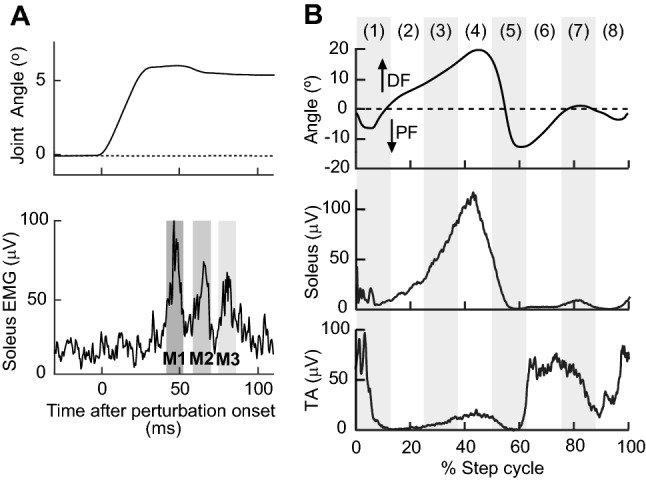
Examples of stretch reflexes, locomotor EMG activity, and ankle joint motion in a participant without known neurological injuries. **a***M*1, *M*2, and *M*3 soleus stretch reflexes elicited by a rapid 6° ankle dorsiflexion (250°/s) during standing. **b** Ankle joint motion and soleus and TA EMG activity during walking in a normal subject. With eight equal bins of the step cycle starting from foot contact, bins 1–4 correspond to the stance phase, bin 5 to the stance-swing transition, and bins 6–8 to the swing phase

At the beginning of the experiment, electromyography (EMG) recording and tibial nerve stimulating electrodes were placed over the leg, and the soleus *H*-reflex/M-wave recruitment curve was obtained, while the participant stood and maintained a stable level of soleus background EMG activity (see EMG recording and nerve stimulation). Then, stretch reflexes were elicited by ankle dorsiflexion (6° at 250°/s) during standing with the same stable level of background EMG activity. Following that, locomotor stretch reflexes were measured, while the participant walked on the treadmill at his/her comfortable speed while wearing a portable joint perturbation device (Andersen and Sinkjaer 1995). Ten-to-fifteen dorsiflexions (aimed at 6°–8° at ≈ 250°/s) were applied in each of 8 equal time bins of the step cycle (Fig. 1). Before or after locomotor stretch reflex measurements, the locomotor *H*-reflex was also measured; soleus *H*-reflexes were elicited by tibial nerve stimulation at just above M-wave threshold in each of 8 equal time bins of the step cycle (as for the stretch reflex measurement). A typical experiment took ~ 1.5 h.

### Device to induce stretch reflexes

To induce and measure stretch reflexes, we used a unique joint motion perturbation device that is capable of imposing a fast ankle joint rotation during gait (Andersen and Sinkjaer 1995). This is a two-link system consisting of a mechanical joint which is strapped to the lower leg and foot of the participant. The mechanical joint aligned to the ankle joint rotates through Bowden wires and is connected to a motor placed next to the treadmill on which the participant walks. Using position feedback from the mechanical joint, the motor can dorsiflex the ankle at a specific ankle joint angle. The system is designed to impose a fast joint rotation of up to 20°. This allows, for example, an 8° joint rotation over 30 ms [known to be effective in inducing stretch reflexes (Sinkjaer et al. 1996a, 1999)] to be performed at any point in the gait cycle. The mechanical joint attached to the participant’s leg and foot weighs < 0.9 kg, and is not too heavy for ambulatory individuals who are capable of treadmill walking (Sinkjaer et al. 1996a; Nielsen et al. 1998; Andersen and Sinkjaer 1999; Turner et al. 1999; Mrachacz-Kersting et al. 2004; Willerslev-Olsen et al. 2014). Indeed, despite wearing the mechanical joint, the participants of this study often did not notice the small rapid ankle dorsiflexions applied during walking. This unique joint motion perturbation device (Andersen and Sinkjaer 1995) has been used safely and effectively in many previous studies, and has revealed the activity of stretch reflex pathways during walking in neurologically normal individuals (Sinkjaer et al. 1996a; Andersen and Sinkjaer 1999; Christensen et al. 2000; Sinkjaer et al. 2000; Grey et al. 2001, 2002; Mazzaro et al. 2005) and individuals with multiple sclerosis (Sinkjaer et al. 1996b, 1999), stroke (Nielsen et al. 1998), and cerebral palsy (Willerslev-Olsen et al. 2014).

### EMG recording and nerve stimulation

The soleus and its antagonist tibialis anterior (TA) EMG signals were recorded with surface self-adhesive Ag–AgCl electrodes (2.2 × 3.5 cm, Vermed, Inc., Bellows Falls, VT). Pairs of EMG recording electrodes were placed just below the gastrocnemius in line with the Achilles tendon for the soleus, and over the muscle belly for the TA, with their centers 3 cm apart. EMG activity was amplified, bandpass filtered (10–1000 Hz), sampled at 4000 Hz, and stored. One participant who wore an ankle foot orthosis removed it for the reflex measurements.

To elicit the soleus *H*-reflex, the tibial nerve was stimulated in the popliteal fossa, using surface Ag–AgCl electrodes (2.2 × 2.2 cm for the cathode electrode and 2.2 × 3.5 cm for the anode electrode, Vermed, Inc., Bellows Falls, VT). The stimulating electrode pair was placed so as to minimize the *H*-reflex threshold, maximize the maximum M-wave size, and avoid stimulation of other nerves.

To elicit the *H*-reflex and M-wave during standing, 1-ms square stimulus pulses were delivered through a Grass S48 stimulator with an SIU-5 stimulation isolation unit and a CCU1 constant current unit (Natus Neurology—Grass, Warwick, RI) when the participant maintained soleus EMG activity within the specified range [i.e., natural standing level of EMG activity, which typically corresponds to 10–20% of maximum voluntary contraction level of EMG activity (Thompson et al. 2009a)] and the resting level of TA activity (i.e., < 7 μV) for at least 2 s. The minimum interstimulus interval was 5 s. To obtain the soleus *H*-reflex/M-wave recruitment curve, the tibial nerve stimulus intensity was varied in increments of 1.2–2.5 mA from the *H*-reflex threshold to the maximum *H*-reflex (*H*_max_) to an intensity just above what was needed to elicit the maximum M-wave (*M*_max_) (Zehr and Stein 1999a; Kido et al. 2004a). Four EMG responses were averaged to measure the *H*-reflex and M-wave at each intensity, and ~ 10 different intensities were used to obtain the recruitment curve.

To elicit stretch reflexes during standing, 6° of rapid dorsiflexion was applied at 250°/s in the same standing posture as the *H*-reflex testing, with the same soleus and TA background EMG level requirements and the same minimum interstimulus interval.

For reflex measurements during walking, foot-switch cells were inserted between the participant’s shoe and foot to detect the foot contact of the stimulated leg. Prior to testing reflexes during walking, the participant walked on a treadmill without stimulation for 1–2 min at his/her comfortable speed (wearing a safety harness) to become used to walking with the mechanical joint attached. As noted above, the device did not cause discomfort or restrict the natural joint motion during walking (except when a perturbation was applied). Thus, the device did not correct or otherwise affect any gait abnormalities such as foot drop. Once the participant was comfortable with the procedure, locomotor reflex testing began. Walking speed, which was self-selected differed (*p* = 0.01, unpaired *t* test) between participants without injuries [0.91 ± 0.05(mean ± SE) m/s] and participants with SCI (0.62 ± 0.08 m/s).

For *H*-reflex testing, single 1-ms square pulse stimuli were delivered at different points in the step cycle (Capaday and Stein 1986; Stein and Capaday 1988; Schneider et al. 2000; Ethier et al. 2003; Kido et al. 2004a). The stimulus interval was long enough to ensure at least one full unstimulated step between stimuli. For analysis, the foot-contact signal was used to define the beginning of the step cycle; thus, the step cycle went from the beginning of the stance phase to the end of the swing phase. EMG signals from individual unstimulated steps (typically > 100 steps) were normalized to the mean step cycle time and averaged to obtain the locomotor EMG activity over the step cycle (Kido et al. 2004b; Makihara et al. 2012; Thompson et al. 2013b; Makihara et al. 2014). The step cycle was divided into 8 equal bins and EMG activity was averaged for each bin. To measure the background EMG activity for *H*-reflex measurement during walking, the average soleus EMG activity was computed for unstimulated steps and used as the control background EMG (Yang and Stein 1990; Kido et al. 2004b; Makihara et al. 2012). The mean rectified *H*-reflex was calculated as [stimulated EMG − control EMG (of the corresponding time in the step cycle)] in the *H*-reflex window (typically 30–45 ms after the stimulus). Then, the sizes of the peak-to-peak or mean rectified *H*-reflexes were plotted vs. the time of the step cycle at which the *H*-reflex was elicited, sorted into 8 equal time bins of the step cycle, and averaged for each bin (Zehr et al. 1997, 1998a; Kido et al. 2004b; Makihara et al. 2012).

For stretch reflex testing, a rapid 6°–8° of ankle dorsiflexion was produced by the attached device every 4–6 steps. Dorsiflexion perturbations occurred in 8 equal bins of the step cycle: they centered at about 5, 17.5, 30, 42.5, 55, 67.5, 80, and 92.5% of the step cycle (Fig. 1). Note that after imposing dorsiflexion perturbation, the mechanical joint was temporarily locked in the post-perturbation position for ≈ 150 ms, to prevent potential contamination of stretch reflex EMG signals by unlocking of the mechanical joint too soon. This temporally affected gait mechanics in the perturbed step but their normalcy was restored within the following step or two (i.e., before the subsequent perturbation was applied). Ten-to-fifteen dorsiflexion perturbations were applied in each bin. The perturbation device system setting that produced ≈ 250°/s rotation during standing resulted in different rotation speeds during walking; actual ankle rotation speeds (summarized in Table 2) were 273 ± 11 (SD)°/s in participants without injuries and 235° ± 10°/s in participants with SCI, different between the groups (*p* < 0.0001, unpaired *t* test). Note that this difference may partly be due to the difference in intrinsic joint stiffness between the groups (Mirbagheri et al. 2001). To determine reflex size, the average EMG activity was calculated for > 50 unperturbed steps and subtracted from the EMG activity of each of the perturbed steps. From the results, *M*1, *M*2, and *M*3 reflex sizes were calculated for each perturbed step, and average *M*1, *M*2, and *M*3 reflex sizes were calculated for each time bin of the step cycle.

**Table 2:** Ankle dorsiflexion perturbation speeds (mean ± SE, °/s) in 8 bins of the step cycle during walking

	Bin 1 @5%	Bin 2 @17.5%	Bin 3 @30%	Bin 4 @42.5%	Bin 5 @55%	Bin 6 @67.5%	Bin 7 @80%	Bin 8 @92.5%
Participants without SCI	283 ± 13	267 ± 7	264 ± 6	264 ± 12	288 ± 8	284 ± 13	271 ± 14	260 ± 10
Participants with SCI	223 ± 11	228 ± 7	222 ± 9	231 ± 9	244 ± 14	244 ± 7	247 ± 8	240 ± 5

The aimed timing of perturbation in % of the step cycle (i.e., from foot contact to foot contact) is indicated for each bin

### Data analysis

To determine the degree to which each muscle’s EMG activity was modulated during walking, the modulation index was calculated in percent as: 100 × [(highest bin amplitude − lowest bin amplitude)/highest bin amplitude] (Zehr and Kido 2001; Kido et al. 2004a, b; Zehr and Loadman 2012; Makihara et al. 2014).

Reflex size was calculated in reference to each participant’s *M*_max_ measured during natural standing. For example, the peak-to-peak *H*-reflex amplitude was normalized to the peak-to-peak *M*_max_ amplitude, and the mean rectified *M*1 amplitude was normalized to the mean rectified *M*_max_ amplitude. To compare the *H*-reflexes elicited at the same effective stimulus strength (i.e., the same M-wave size) across different phases of the step cycle, various stimulus intensities were used (Capaday and Stein 1986; Llewellyn et al. 1990; Edamura et al. 1991); and only the *H*-reflexes with comparable M-wave sizes were included in the analysis. Note that, in the present study, we did not elicit the *M*_max_ at each part of the step cycle, to avoid potential negative effects of *M*_max_ eliciting high intensity stimuli (e.g., discomfort and fatigue) altering locomotor EMG and kinematics. While the soleus *M*_max_ amplitude could vary during walking (Simonsen and Dyhre-Poulsen 1999; Ferris et al. 2001), its range can remain close to that measured during natural standing (e.g., see Fig. 5 of Kido et al. 2004a). Thus, in this study, all the reflex size and M-wave size that accompanied the *H*-reflex were normalized to the *M*_max_ during standing (Thompson et al. 2013a; Makihara et al. 2014). Typically, 10–15 responses were averaged in each of the 8 bins for both the *H*-reflex and stretch reflexes.

For comparing phase-dependent modulation of reflexes in two different groups, a two-way repeated measures ANOVA (groups × step cycle bins) was used, together with Student *t* test with Bonferroni correction as a post hoc test (i.e., for comparison between step cycle bins, *α* = 0.05, *p* < 0.0063 and *α* = 0.1, *p* < 0.0125). For simple comparison between participants without injuries and participants with SCI (e.g., age and *M*_max_), a Student *t* test was used.

In addition, for assessing the relationship between the *H*-reflex and stretch reflexes during walking, Pearson’s correlation coefficient (*r* value) was calculated between *H*-reflex size (in %*M*_max_) and stretch reflex size (in %*M*_max_) across the step cycle in each participant, and for each of the 8 step cycle bins across participants. We also performed correlation analysis on locomotor background (control) EMG (i.e., non-stimulated steps’ EMG of the corresponding time in the step cycle) and locomotor *H*-, *M*1, *M*2, and *M*3 reflex sizes.

To assess the impact of stretch or *H*-reflex activity on locomotor EMG in the subsequent step, soleus EMG was averaged for an extended post-stimulus (or perturbation) period that equaled the duration of the step cycle. Then, the number of clonus-like bursts that followed stretch or *H*-reflex (or reflex-eliciting stimulation/perturbation in the absence of reflex activity) was determined in each bin for each participant. Rhythmic bursts at 5–8 Hz [i.e., with 125–200 ms intervals (Latash et al. 1989; Rossi et al. 1990; Wallace et al. 2005; Gorassini et al. 2009; Wallace et al. 2012)] with peak amplitudes exceeding 1.5 × unstimulated step EMG amplitudes of the corresponding times in the step cycle were counted as clonic bursts.

## Results

### ***H*-**reflex and stretch reflexes during standing

Soleus *H*-reflex and stretch reflexes were measured, while the participant maintained a natural standing posture and kept soleus and TA EMG activity within the specified ranges. The absolute soleus background EMG amplitude was similar between participants without injuries and participants with incomplete SCI; 17 ± 2 (mean ± SE) μV for participants without injuries and 16 ± 2 μV for participants with SCI (*p* = 0.96, unpaired *t* test). When normalized to *M*_max_ (i.e., mean rectified *M*_max_), it was 1.4 ± 0.2%*M*_max_ for participants without injuries and 1.6 ± 0.3%*M*_max_ for participants with SCI, and there was no significant difference (*p* = 0.56, unpaired *t* test). TA activity remained at the resting level, (i.e., < 7 μV) in both groups of participants. Thus, the background (pre-stimulus) EMG profiles during standing were very similar between these groups of participants.

*M*_max_ and *H*_max_ did not differ significantly between the groups. Peak-to-peak *M*_max_ was 8.0 ± 1.6 (mean ± SE) mV in participants without injuries and 5.5 ± 0.7 mV in participants with SCI (*p* = 0.19), and the peak-to-peak *H*_max_ was 3.2 ± 1.4 mV in participants without injuries and 2.8 ± 0.8 mV in participants with SCI (*p* = 0.83). The resulting *H*_max_/*M*_max_ ratio was not significantly different (*p* = 0.33); 0.35 ± 0.06 for participants without injuries and 0.46 ± 0.08 for participants with SCI. Mean rectified *H*_max_ was 32 ± 5 (mean ± SE)%*M*_max_ in participants without injuries and 51 ± 13%*M*_max_ in participants with SCI, not significantly different between the groups (*p* = 0.19).

For the soleus stretch reflexes, centers of responses (i.e., peak latencies) for the *M*1, *M*2, and *M*3 were similar between the groups: 56.8 ± 1.1 (mean ± SE) ms, 73.7 ± 1.0 ms, and 88.6 ± 0.3 ms for *M*1, *M*2, and *M*3 in participants without injuries; and 54.3 ± 0.5 ms, 72.1 ± 0.6 ms, and 88.8 ± 0.7 ms for *M*1, *M*2, and *M*3 in participants with SCI (*p* > 0.07 for *M*1, *M*2, and *M*3). In contrast, the stretch reflex sizes differed significantly between the groups. Mean rectified *M*1, *M*2, and *M*3 were 3.3 ± 0.6 (mean ± SE)%*M*_max_, 2.4 ± 0.4%*M*_max_, and 2.9 ± 0.6%*M*_max_ in participants with SCI; they were larger than those in participants without injuries (1.7 ± 0.2%*M*_max_, 1.8 ± 0.3%*M*_max_, and 1.4 ± 0.3%*M*_max_ for *M*1, *M*2, and *M*3, respectively) (*p* = 0.02 for *M*1, *p* = 0.31 for *M*2, and *p* = 0.03 for *M*3, unpaired *t* test). Comparisons of the peak-to-peak *M*1, *M*2, and *M*3 values between the groups showed similar trends, but did not yield significant differences (*p* > 0.26 for *M*1, *M*2, and *M*3): in participants with SCI vs. participants without injuries 2.9 ± 0.5 vs. 2.3 ± 0.3%*M*_max_ for *M*1, 2.3 ± 0.4 vs. 2.6 ± 0.3%*M*_max_ for *M*2, and 2.6 ± 0.4 vs. 1.9 ± 0.4%*M*_max_ for *M*3.

### *H*-reflex during walking

**Fig. 2:**
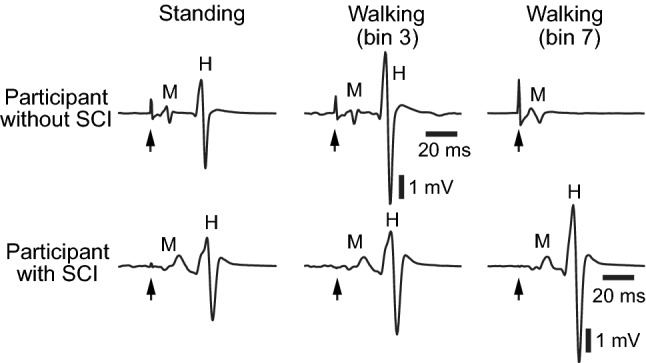
Examples of the soleus *H*-reflex during standing and walking in a participant without known neurological injuries (top) and in a participant with chronic incomplete SCI (bottom). Height, weight, and age are similar in these two individuals. More than 10 EMG sweeps were averaged for each panel. For each trace, the stimulus is indicated (with arrow) and the M-wave and *H*-reflex are labeled. In the participant without injuries, the soleus *H*-reflex was large in the mid-stance phase (i.e., bin 3) when the muscle was active and contributing to functionally meaningful force generation, and was completely suppressed in the mid–late-swing phase (i.e., bins 7 and 8) when the muscle was not active. In the participant with SCI, the *H*-reflex was large in the mid–late swing, in stark contrast to the normal subject

Typical examples of soleus *H*-reflexes during standing and walking are shown in Fig. 2. The top row displays *H*-reflexes in a participant without injuries: during standing with the pre-defined natural standing level of soleus EMG activity; during the mid-stance phase of walking (i.e., bin 3); and during the mid–late-swing phase of walking (i.e., bin 7). The M-waves that accompany these *H*-reflexes were all similar in size. As documented previously (Capaday and Stein 1986; Kido et al. 2004a; Makihara et al. 2012), the soleus *H*-reflex was dynamically and phase-dependently modulated during walking. The amplitude was high in the mid-stance phase (i.e., bin 3) when the muscle was active and contributing to functionally meaningful force generation, whereas in the mid–late-swing phase (i.e., bins 7 and 8), when the muscle was not active [and its contraction could be harmful (i.e., causing tripping)], the amplitude was at its minimum (Capaday and Stein 1986; Stein and Capaday 1988). In contrast, in a participant with chronic incomplete SCI (bottom row), the *H*-reflex was large in the mid–late swing (bins 7 and 8), during which the participant without injuries showed no *H*-reflex.

**Fig. 3:**
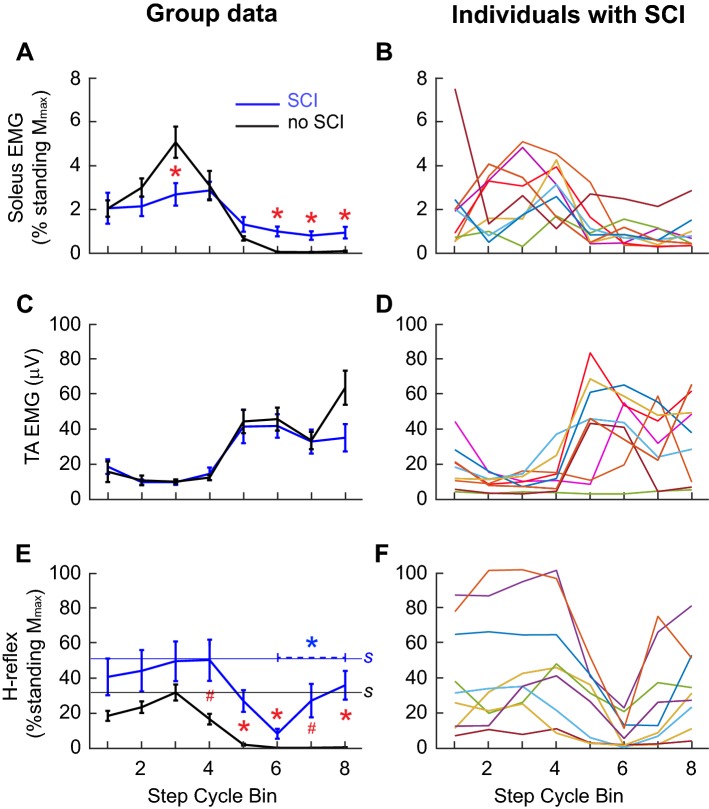
Soleus and TA EMG activity and *H*-reflexes, during walking in participants with chronic incomplete SCI (*N* = 9, mean ± SE) and participants without injuries (*N* = 9, mean ± SE). **b**, **d**, **f** Values for each individual with SCI. For calculating *H*-reflex size, for each participant, ≥ 10 EMG sweeps in each of eight equal bins of the step cycle were averaged together and normalized to *M*_max_. In general, bins 1–4 correspond to the stance phase, bin 5 to the stance-swing transition, and bins 6–8 to the swing phase. **a**, **b** Soleus EMG in control (unperturbed) steps. More than 100 control steps were averaged for each subject. **c**, **d** TA EMG amplitude in μV. **e**, **f** Soleus mean rectified *H*-reflex in % standing *M*_max_. Horizontal lines labeled with “*S*” in **e** indicate the average *H*_max_ sizes during standing. For all panels, statistically significant differences between the groups and between the bins (in participants with SCI only) by Student *t* test with Bonferroni correction are indicated with * (*α* = 0.05, *p* < 0.0063) and # (*α* = 0.1, *p* < 0.0125)

Soleus and TA EMG and *H*-reflex modulation over the step cycle (group mean ± SE) is shown in Fig. 3. We measured both peak-to-peak and mean rectified values for the *H*-reflex and stretch reflexes (next section). Since both modes of analysis yielded similar results, only mean rectified values are presented in here and in the sections below. Although the number of step cycle bins used in this study (i.e., 8) was smaller than in some previous studies of locomotor reflex modulation (Hase and Stein 1998; Zehr et al. 1998a, b; Kido et al. 2004b), it was sufficient to delineate phase-dependent modulation (as it did in Sinkjaer et al. 1996a, 1999; Zehr and Loadman 2012). In participants with SCI, soleus EMG during control (i.e., unperturbed) steps was less modulated than participants without injuries (*p* < 0.0001 by unpaired *t* test) (Fig. 3a, Table 3). Similarly, *H*-reflex modulation over the step cycle was less in participants with SCI than in participants without injuries (*p* = 0.002). The differences between the two groups in *H*-reflex amplitude and modulation was confirmed by a two-way (groups × step cycle bins) repeated measures ANOVA, which indicated significant effects of groups (*p* = 0.02), bins (*p* < 0.0001), and their interaction (*p* = 0.02). Notably, the *H*-reflex was larger in participants with SCI than in participants without injuries in bins 4–8 (i.e., the mid–late stance phase through the late-swing phase). In Fig. 3e, these differences (by Student *t* test with Bonferroni correction) are indicated by * (*α* = 0.05, *p* < 0.0063) and # (*α* = 0.1, *p* < 0.0125). In addition, in participants with SCI, the *H*-reflex in bin 8 (late-swing phase) was significantly larger than that in bin 6 (mid-swing phase) (Fig. 3e), indicating that *H*-reflex modulation was not only reduced but also was abnormal in form in the swing phase of the step cycle.

**Table 3:** Modulation index (mean ± SE) over the step cycle

	Soleus EMG	TA EMG	*H*-reflex	*M*1	*M*2	*M*3
Participants without SCI	98.8 ± 0.2	83.5 ± 2.6	99.5 ± 0.1	96.0 ± 1.5	90.7 ± 3.4	88.5 ± 5.1
Participants with SCI	86.3 ± 2.1*	81.7 ± 5.0	85.2 ± 4.2*	89.5 ± 2.9*	95.3 ± 2.0	78.6 ± 9.2

Modulation index was calculated in percent as: 100 × [(highest bin amplitude − lowest bin amplitude)/highest bin amplitude] (Zehr and Kido 2001; Kido et al. 2004a, b; Zehr and Loadman 2012; Makihara et al. 2014)

*Statistically significant difference between the normal subjects and subjects with incomplete SCI (unpaired *t* test, *p* < 0.05)

TA EMG was modulated over the step cycle in both participants without injuries and participants with SCI (Table 3, Fig. 3c, d), and its modulation index did not differ between the groups.

### Stretch reflexes during walking

**Fig. 4:**
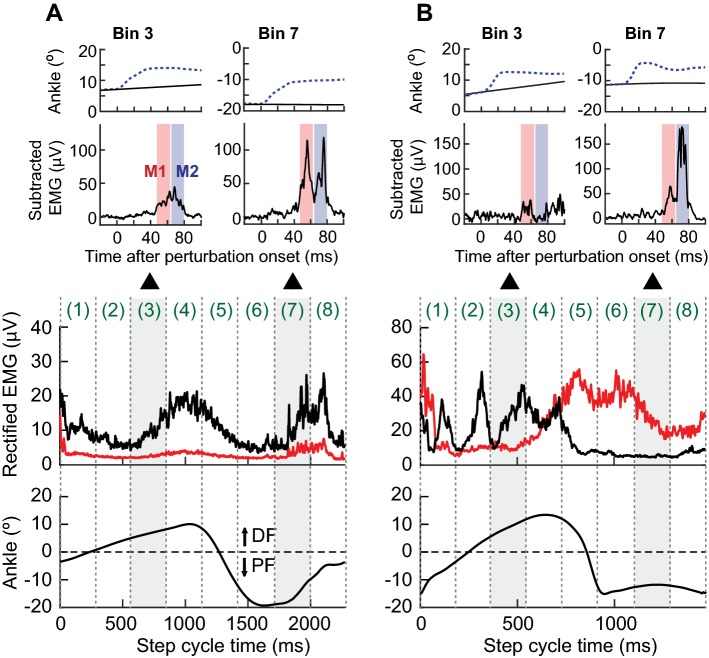
Soleus stretch reflexes elicited by 6° dorsiflexion perturbation at 250°/s in bin 3 and 7 of the step cycle and soleus and TA EMG and ankle joint motion during walking in two representative individuals (**a**, **b**) with chronic incomplete SCI. In each individual, the top panel shows the average ankle joint motion around the time of perturbation in unperturbed (i.e., control) steps (solid line) and perturbed steps (dotted line). The second panel shows the averaged [perturbed − unperturbed] EMG response. Ten-to-fifteen EMG responses were averaged together. The third panel shows locomotor EMG activity in the soleus (black) and TA (red). The fourth panel shows the average ankle joint motion of unperturbed steps **(**averaged over > 50 control steps)

Figure 4 shows typical examples of soleus and TA EMG and ankle joint motion during walking, and soleus stretch reflexes in bin 3 and 7 of the step cycle in participants with chronic incomplete SCI. The top panels show the average ankle joint motion around the time of perturbation in control (i.e., unperturbed) steps (solid line) and perturbed steps (dotted line). Panels in the second row show the averaged [perturbed–unperturbed] EMG sweeps. Thus, the excitation caused by the perturbation appears as additional EMG activity. Panels in the bottom half are for locomotor EMG activity in the soleus (black line) and TA (red line) and ankle joint motion over the entire step cycle, obtained by averaging > 50 control (i.e., unperturbed) steps. Locomotor EMG activity and ankle joint motion in these participants were clearly different from those in participants without injuries (see Fig. 1b). In the participant of Fig. 4a, there was a noticeable burst of activity in the soleus during the swing phase (note that in this participant the toe dragged throughout the swing phase and never came off the ground), while there was little TA activity. In bin 3, the absolute sizes of *M*1 and *M*2 were 33 μV and 28 μV, similar to those of participants without injuries (mean rectified *M*1 and *M*2 are 27 ± 11 μV and 26 ± 4 μV, respectively, in participants without injuries); but in bin 7, in which participants without injuries exhibit little stretch reflex activity (i.e., < 2.5 μV), both *M*1 and *M*2 were clearly larger (69 μV and 80 μV, respectively). In the participant of Fig. 4b, there was no dorsiflexion throughout the swing phase, despite the presence of some TA activity in the early–mid-swing phase. In the soleus, clonus was present in the early–mid-stance phase and disappeared in the swing phase. Similar to the observation in the participant of Fig. 4a, *M*1 and *M*2 in bin 3 were not remarkable; however, in bin 7, they were strikingly clear and large (42 μV and 119 μV, respectively) in the absence of ongoing soleus EMG activity.

**Fig. 5:**
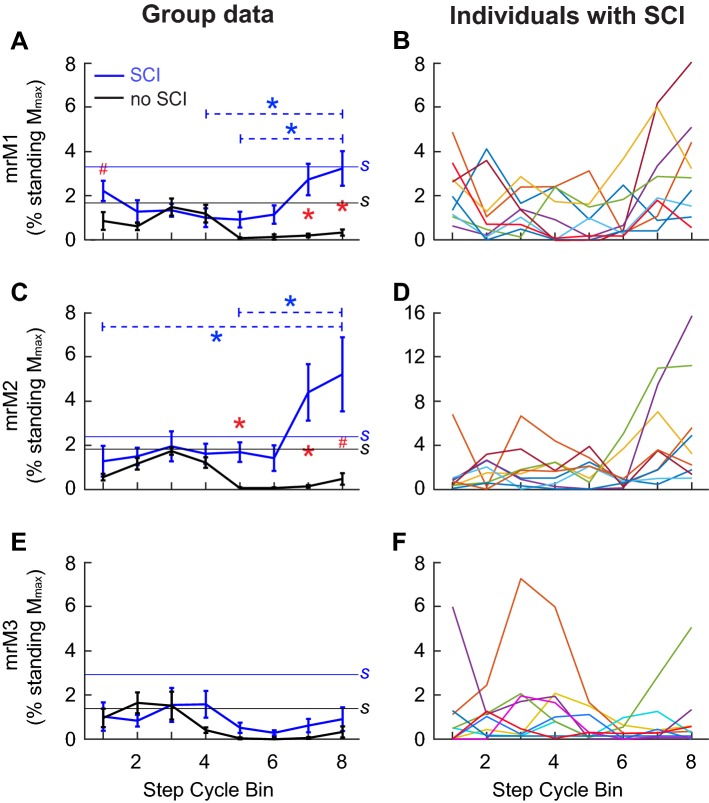
Soleus stretch reflexes during walking in participants with chronic incomplete SCI (blue in **a**, **c**, and **e**, *N* = 9, mean ± SE) and participants without injuries (black in **a**, **c**, and **e**, *N* = 9, mean ± SE). For each participant, ≥ 10 EMG responses in each of eight equal bins of the step cycle were averaged together and normalized to *M*_max_. In general, bins 1–4 correspond to the stance phase, bin 5 to the stance-swing transition, and bins 6–8 to the swing phase. *M*1 (**a**, **b**), *M*2 (**c**, **d**), and *M*3 (**e**, **f**) reflex sizes are calculated as mean rectified values. In **a**, **c**, and **e**, horizontal lines labeled with “*S*” indicate the average *M*1, *M*2, or *M*3 sizes during standing (the blue line is for the group with SCI, and the black line is for the group without injuries). **b**, **d**, **f** Values for each individual with SCI. For all panels, statistically significant differences between the groups and between the bins (in participants with SCI only) by Student *t* test with Bonferroni correction are indicated with * (*α* = 0.05, *p* < 0.0063) and # (*α* = 0.1, *p* < 0.0125)

Soleus stretch reflex modulation over the step cycle (group mean ± SE) is shown in Fig. 5. In participants with SCI, *M*1 modulation was significantly less than in participants without injuries (*p* = 0.04, unpaired *t* test) (Fig. 5a, Table 3). Although the patterns differed from those in participants without injuries, *M*2 and *M*3 were modulated in participants with SCI; their extents of modulation were not significantly different from those of normal participants (*p* > 0.08 for both measures) (Table 3).

The differences in stretch reflex modulation and reflex amplitudes between the groups were further evaluated by a two-way (groups × step cycle bins) repeated measures ANOVA. For *M*1, the effect of groups (*p* = 0.0025), bins (*p* = 0.0075), and their interaction (*p* = 0.0004) were all significant. The post hoc analysis (Student *t* test with Bonferroni Correction) indicated that, in bins 7 and 8 (i.e., mid–late-swing phase), *M*1 was significantly larger in participants with SCI than in participants without injuries and that in participants with SCI, *M*1 in the late-swing phase was larger than that in late stance (Fig. 5a). These results suggest that *M*1 modulation is not only reduced, but is also abnormal in participants with SCI. The effect of groups (*p* = 0.004), bins (*p* = 0.006), and their interaction (*p* = 0.0008) were all significant for *M*2 modulation. Post-hoc tests indicated that, in bins 5, 7, and 8, *M*2 was significantly larger in participants with SCI than in participants without injuries; and that, in participants with SCI, *M*2 was larger in the late-swing phase than in the late stance or early swing phase (Fig. 5c). The general features of *M*2 modulation in participants with SCI were similar to those of *M*1. During the stance phase, *M*2 was not notably different from that in participants without injuries. However, in the mid–late-swing phase, the difference between the groups became very clear: *M*2 was suppressed in participants without injuries and enhanced in participants with SCI. For *M*3, the effect of bins was significant (*p* = 0.004), as expected from the modulation index analysis above. However, the effect of groups and the groups × bins interaction were not significant (*p* = 0.49 and 0.35, respectively). Overall, the modulation of *M*3 was not markedly different between participants with SCI and participants without injuries (Fig. 5e). In Fig. 5, differences detected by Student *t* test with Bonferroni Correction are indicated by * (*α* = 0.05, *p* < 0.0063) and # (*α* = 0.1, *p* < 0.0125).

### Relations between background EMG, *H*-reflex and stretch reflexes during walking

**Fig. 6:**
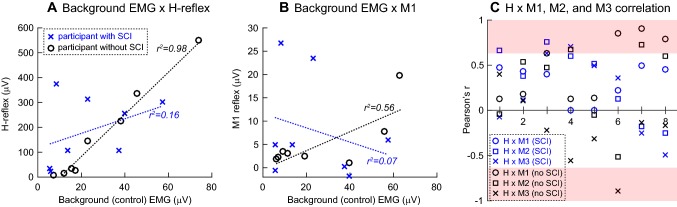
Correlation between the soleus locomotor background EMG and *H*-reflex (**a**) or *M*1 stretch reflex size (**b**) and correlation between *H*-reflex size and *M*1, *M*2, or *M*3 stretch reflex size (**c**). **a** Mean rectified *H*-reflex sizes from 8 bins of the step cycle are plotted against background EMG (i.e., control EMG of non-stimulated steps for the period corresponding to the time of reflex elicitation) in a representative participant with SCI (blue crosses) and a participant without SCI (black circles). Coefficient of determination *r*^2^ values and the corresponding regression lines are also indicated in blue (participant with SCI) and black (participant without SCI). **b** Mean rectified *M*1 reflex sizes from eight bins of the step cycle are plotted against background (control) EMG in the same representative individuals as in **a**. **c** Pearson’s correlation coefficient *r* value is calculated for each of the 8 bins of the step cycle for each group. Light red bands indicate statistically significant (*p* < 0.05) *r* value ranges

To estimate how much of the variance in reflex size would be explained by locomotor background EMG activity, the correlation between background EMG (control EMG of non-stimulated steps for the corresponding time in the step cycle) and *H*, *M*1, *M*2, and *M*3 reflexes were assessed in each participant. As previously shown (e.g., Capaday and Stein 1986), in neurologically normal participants, the size of the locomotor *H*-reflex was strongly correlated with background EMG activity [*r* = 0.91 ± 0.02 (group mean ± SE)], and the *r*^2^ value of 0.84 ± 0.04 indicates that a large portion of reflex size variation is explicable by background EMG activity. *M*1, *M*2, and *M*3 reflex sizes also correlated with background EMG although to a lesser extent (*r* = 0.70 ± 0.07, 0.81 ± 0.04, and 0.48 ± 0.16, respectively), and their variations were partially explainable by background EMG activity (*r*^2^ = 0.52 ± 0.08, 0.66 ± 0.07, and 0.44 ± 0.11, respectively). In participants with SCI, these relations were far weaker for both *H* and stretch reflexes; (*r*^2^ = 0.23 ± 0.05, 0.07 ± 0.02, 0.11 ± 0.03, and 0.26 ± 0.09, for *H*, *M*1, *M*2, and *M*3, respectively). The coefficient of determination (i.e., *r*^2^) differed significantly between the groups of participants with and without SCI for the *H*-reflex (*p* < 0.0001, by two-tailed *t* test), *M*1 (*p* = 0.0008), and *M*2 (*p* < 0.0001), but not for *M*3 (*p* = 0.25). Figure 6a, b exemplifies these relations in representative individuals of both groups.

To assess whether there is a predictable relationship between the sizes of *H*-reflex and stretch reflexes in the soleus, we calculated Pearson’s correlation coefficient (*r* value) across the step cycle in each participant. In participants without SCI, a moderate positive correlation was found for all three components of the stretch reflex; *r* = 0.62 ± 0.07 (group mean ± SE) for *H* × *M*1, *r* = 0.73 ± 0.05 for *H* × *M*2, and *r* = 0.50 ± 0.16 for *H* × *M*3. In contrast, in participants with SCI, the correlation was weak to none for all three components of the stretch reflex; *r* = 0.03 ± 0.12 for *H* × *M*1, *r* = 0.23 ± 0.12 for *H* × *M*2, and *r* = 0.24 ± 0.11 for *H* × *M*3. We also calculated the *r* value for each of the eight-step cycle bins across participants. Figure 6c summarizes the correlation coefficients between *H*-reflex size and *M*1, *M*2, or *M*3 stretch reflex size. In general, there were no obvious trends in the *r* value distribution for any of the three stretch reflex components in either group.

### Effects of reflex elicitation in the late-swing phase of walking

**Fig. 7:**
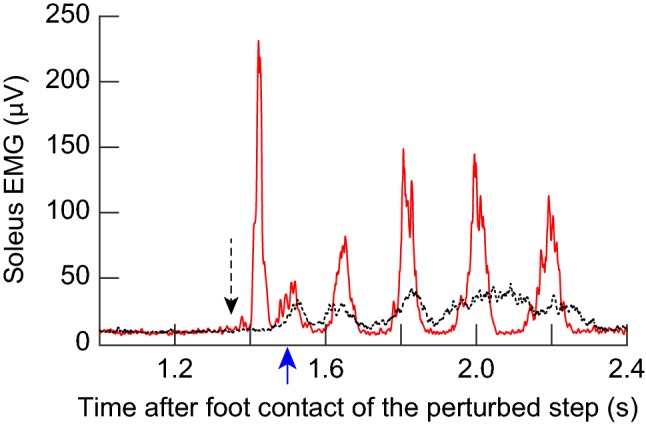
Soleus EMG activity after 7° dorsiflexion perturbation at 240°/s in bin 8 in a participant with SCI. Fifteen perturbed EMG sweeps were averaged together (solid), and 62 unperturbed EMG sweeps over the corresponding phase of step cycle were averaged together (dotted). A black arrow with a dashed line indicates the onset of perturbation. A blue arrow (around 1500 ms) indicates the foot contact of the step immediately after. Perturbation introduced at ≈ 1350 ms after foot contact (i.e., ≈ 150 ms before the following foot contact) elicited large stretch reflexes (*M*1 and *M*2 appear to be fused in this figure, since the horizontal scale displays > 1000 ms after perturbation) and clonic EMG bursts over the subsequent stance phase. Robust clonus replaced the fused mid–late stance EMG activity that was present in unperturbed steps

As summarized in Fig. 3c, tibial nerve stimulation in the mid–late-swing phase (i.e., bins 7 and 8) elicited little or no soleus *H*-reflex in participants without injuries. Similarly, dorsiflexion perturbations applied in the mid–late-swing phase elicited little or no stretch reflexes in participants without injuries (Fig. 5). In contrast, in participants with SCI, *H*-reflex and stretch reflexes were present in this phase, and were often larger than those in the mid–late stance phase (Figs. 3c, 5). To assess the impact of this abnormal reflex activity in participants with SCI, soleus EMG activity was averaged for an extended post-stimulus (or perturbation) period that equaled the duration of the step cycle. Figure 7 shows a typical example of post-stretch reflex EMG activity after a perturbation in bin 8. The averaged perturbed EMG (*N* = 15, solid) is shown with the average unperturbed EMG (*N* = 62, dotted). In this example, the perturbation was introduced at ≈ 1350 ms after foot contact (i.e., late-swing phase). It elicited large stretch reflexes and clonic EMG bursts over the subsequent stance phase; clonus was so robust that it replaced the fused EMG activity that was present during unperturbed stance. Similar induction and/or amplification of clonic bursts in the immediately following step were observed after perturbations in bin 8 in all 9 participants with SCI studied here (Fig. 9a). In clear contrast, in participants without injuries, elicitation of stretch reflexes or reflex-eliciting dorsiflexion perturbations rarely triggered any discernable clonic bursts across the step cycle (Fig. 9c).

**Fig. 8:**
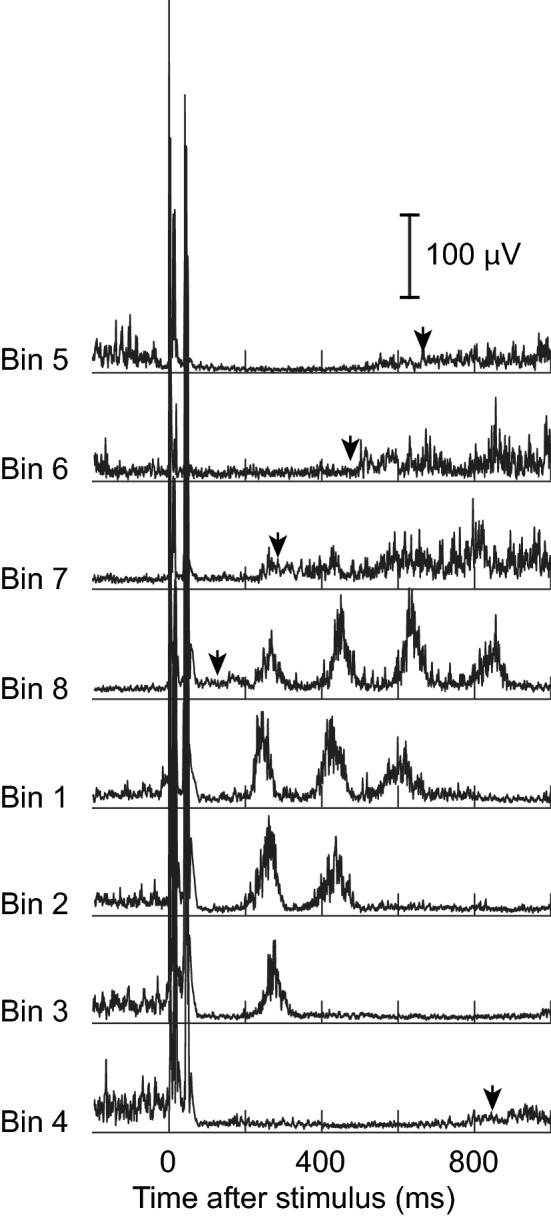
Soleus EMG activity after *H*-reflex-eliciting tibial nerve stimulation in 8 bins of the step cycle in a participant with SCI. In each panel, 11–15 EMG sweeps following the *H*-reflexes with similar M-wave sizes were averaged together. Black arrow heads indicate the times of foot contact in the steps immediately after stimulation. *H*-reflex elicitation in bins 8 (late swing) and bins 1 and 2 (early and mid-stance) led to several clonic bursts that replaced the fused mid–late stance push-off burst

**Fig. 9:**
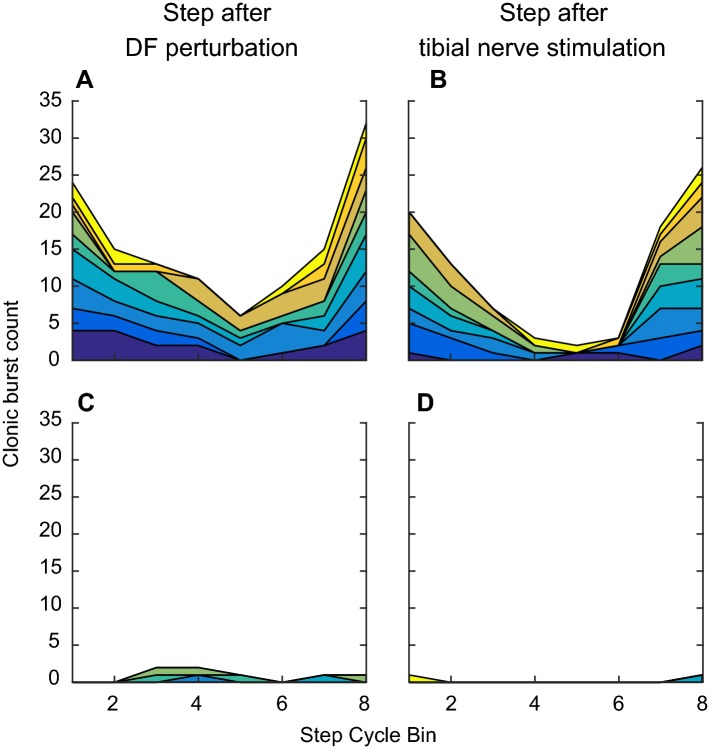
Counts of clonus-like rhythmic bursts in the locomotor soleus EMG activity after dorsiflexion perturbation (**a**, **c**) or *H*-reflex-eliciting tibial nerve stimulation (**b**, **d**) in 8 bins of the step cycle in participants with SCI (**a**, **b**) and in participants without injuries (**c**, **d**). Each color represents a single participant. In participants with SCI, dorsiflexion perturbation or *H*-reflex-eliciting tibial nerve stimulation often triggered clonus-like (i.e., 5–8 Hz burst) in bins 7, 8, 1, and 2. In contrast, such phasic bursts were rare across participants without injuries at any phases of the step cycle

Figure 8 shows the EMG activity after tibial nerve stimulation in 8 bins of the step cycle in a typical participant with SCI. Eleven-to-fifteen EMG sweeps are averaged for each bin. It is clear that *H*-reflex elicitation in bins 4, 5, and 6 (i.e., mid–late stance phase and early swing phase) did not induce clonus, whereas in bins 8 (late swing) and 1 (early stance), it triggered clonic bursts. Interestingly, this induction of clonus or clonus-like phasic EMG bursts [of 5–8 Hz, (Latash et al. 1989; Rossi et al. 1990; Wallace et al. 2005; Gorassini et al. 2009; Wallace et al. 2012)] by *H*-reflex elicitation was a phase-dependent phenomenon. The number of clonic bursts after *H*-reflex elicitation progressively declined from bin 8 to bin 3; clonus appeared to cease in the late stance phase, regardless of how many bursts had been produced up to that point. Across all SCI participants of this study, we observed that *H*-reflex-eliciting tibial nerve stimulation in bin 8 triggered clonus or clonus-like burst(s) of rhythmic excitation, whereas the same stimulation in bins 4 and 5, despite producing larger *H*-reflexes, did not trigger similar rhythmic bursts (Fig. 9b). In participants without injuries, elicitation of the *H*-reflex or the same relative intensity of tibial nerve stimulation that elicited the *H*-reflex in the stance phase rarely triggered any discernable clonic bursts across the step cycle (Fig. 9d). Exploration of the physiological mechanisms underlying these observations was beyond the scope of this study. Further studies are needed to understand the mechanisms of clonus and its susceptibility to spinal and supraspinal inhibition in people with chronic SCI.

## Discussion

This study examined soleus *H*-reflexes and stretch reflexes during standing and walking in people with spasticity due to chronic incomplete SCI. During standing, the differences in sizes of soleus *H*-reflex and stretch reflexes between participants with and without SCI were small and often statistically insignificant. Such insignificant differences were surprising, as higher *H*_max_/*M*_max_ ratios and exaggerated stretch reflexes have been reported in spastic participants with SCI and other CNS disorders previously (Taylor et al. 1984; Brown 1994; Thompson et al. 2009b). This could be partly explained by baclofen [which increases the stretch reflex threshold in people with spasticity due to multiple sclerosis (Nielsen et al. 2000; Nielsen and Sinkjaer 2000)] and/or by other anti-spastic medications that our participants with SCI were taking. In addition, several inhibitory mechanisms acting on these spinal reflexes would be reduced in actively contracting muscles in participants with and without spasticity (Nielsen et al. 2005; Dietz and Sinkjaer 2012, 2015), and thus, might have diminished the differences between participants without injuries and participants with SCI.

Nevertheless, modulation of the *M*1 and *M*2 stretch reflexes and *H*-reflex was clearly abnormal during walking in participants with SCI. Normal positive correlation between locomotor background EMG and reflex excitability was rarely found in those individuals. An important implication of these observations is that reflexes elicited in selected passive states, during isolated joint movements, and/or during static motor tasks do not necessarily indicate how the reflexes function or malfunction during specific phases of dynamic motion, such as walking. Here, we found that the excitability of spinal stretch reflex pathways was abnormally high from the stance-swing transition through the late-swing phase in ambulatory individuals with chronic incomplete SCI.

### *H*-reflex and stretch reflex modulation during walking in people with chronic incomplete SCI

For stretch reflexes, group Ia and II muscle spindle afferents are excited by stretch of the nerve endings in the spindle, affected by γ-motoneuron mediated fusimotor control, and muscle stretch (tension change) excites group Ib Golgi tendon organ afferents; for the *H*-reflex, group Ia (and some II) muscle spindle afferent axons and group Ib Golgi tendon organ afferent axons are excited electrically (Magladery et al. 1951; Lundberg et al. 1978; Henneman and Mendell 1980; Jankowska and McCrea 1983; McKeon and Burke 1983; Morita et al. 1998; Schafer et al. 1999; Duysens et al. 2000; Jankowska and Edgley 2010; Vincent et al. 2017; Nichols 2018). While some differences between stretch and *H*-reflexes were noted as in earlier studies (Morita et al. 1998; Andersen and Sinkjaer 1999), the present study found that the spinal short-latency *M*1 stretch reflex and the *H*-reflex (both thought to originate mainly from Ia afferents) were both suppressed in the swing phase in participants without injuries but not in participants with SCI (Figs. 3c, 5). This may suggest reduced inhibition of Ia excitatory pathways in this phase in participants with SCI. Modulation of the spinal medium-latency *M*2 reflex, presumably mainly group II afferent-mediated (Corna et al. 1995; Schieppati and Nardone 1997; Nardone and Schieppati 1998; Grey et al. 2001; Af Klint et al. 2010), was similar to that of *M*1 (for both participants with and without SCI), with strikingly large amplitudes in the mid–late-swing phase for participants with SCI (Fig. 5). Exaggerated *M*2 responses in these participants could be due to reduced inhibition of spindle afferent II excitation, increased oligo or polysynaptic Ia excitation, and/or increased excitation of other afferents. Actions of excitatory and inhibitory interneurons that receive input from Ib afferents could also affect *M*2 modulation (Schafer et al. 1999; Jankowska and Edgley 2010). Since our current understanding of Ia, II, and Ib afferent firing patterns and functional contributions of those afferents to muscle activity is largely based on feline studies (e.g., (Jankowska 1979; Jankowska and McCrea 1983; Donelan and Pearson 2004; Jankowska and Edgley 2010; Hatz et al. 2012), it is not clear in humans how those afferents from plantarflexors fire during locomotion and whether and how their input to interneurons result in muscle activation. However, presuming fundamental similarities in the characteristics of those afferents across species, it is possible that spindle afferent II could be firing in the late-swing phase (i.e., pre-stance) (Donelan and Pearson 2004; Hatz et al. 2012) even when there is no EMG activity, and it is likely that Ib and II afferents would be firing when there is EMG activity (Donelan and Pearson 2004; Hatz et al. 2012). These afferent firing behaviors, together with altered modulation or excitability of Ib/II interneurons, may explain abnormal swing phase burst in the soleus EMG (e.g., Fig. 4a) or abnormally large *M*2 response in the late-swing phase (e.g., Fig. 4b) in pathologic gait, at least partly. Clearly, further studies are needed to determine which afferents (or afferent combinations) contribute to *M*2 in individuals with SCI.

The long-latency *M*3 response, which may be a transcortical or subcortical response (Sinkjaer et al. 1999; Christensen et al. 2001; Zuur 2013), was phase-dependently modulated similarly in both groups of participants. Although currently, there is no direct evidence indicating the pathway(s) of *M*3 in participants with SCI, a surprisingly near normal pattern of *M*3 modulation (in contrast to abnormal *M*1 and *M*2 modulation) and the presence of *M*3 in the mid–late stance phase of walking in some participants with incomplete SCI (e.g., Fig. 4b) (in whom some corticospinal connections could remain), supports the possibility that *M*3 is a transcortical response in these participants.

One of the important analyses made in this study is the *H*-reflex—stretch reflex correlation analysis. As mentioned in “Introduction”, although the *H*-reflex is often used to investigate Ia excitation of homonymous motoneurons (Schieppati 1987; Zehr 2002; Misiaszek 2003), *H*-reflex and *M*1 stretch reflex do not necessarily respond to modulatory input in the same way (Nielsen et al. 1994; Sinkjaer et al. 1996a; Morita et al. 1998). In the present study, stretch reflexes and *H*-reflex were measured in the same individuals during the same task of treadmill walking, which provided a unique opportunity for assessing the predictability of stretch reflex excitability from the *H*-reflex measurement. In individual participants without SCI, moderate positive correlation was found between the *H*-reflex and all three components of stretch reflexes across the 8 equal bins of the step cycle, supporting a moderate predictability of stretch reflex behavior from the *H*-reflex measurements during walking in neurologically normal individuals. In contrast, in participants with SCI, such correlation was weak to non-existing, suggesting that the locomotor *H*-reflex amplitudes would not provide reliable estimates of stretch reflex excitability in individuals of this population. Furthermore, across individuals, *H*-reflex size at a given phase of step cycle did not necessarily predict *M*1 (or *M*2 or *M*3) reflex size effectively; as shown in Fig. 6c, *H*-reflex size did not systematically correlate with *M*1, *M*2, or *M*3 stretch reflex size in any of the eight step cycle bins (with potential exceptions in the mid-to-late-swing phase in the group without SCI, in whom the reflexes in these bins are small to begin with (see Figs. 3c, 5). Thus, the present observation suggests that investigation of the *H*-reflex would not substitute for investigation of *M*1, *M*2, or *M*3 stretch reflexes in the soleus, especially in individuals with chronic SCI.

### Etiology of abnormal locomotor reflex modulation in people with incomplete SCI

In individuals without known neurological injuries, the soleus *H*-reflex is task- and phase-dependently modulated during walking. Task-dependent reduction of reflex gain from standing to walking to running is thought to be due to increased presynaptic inhibition (Capaday and Stein 1987b; Stein and Capaday 1988) caused by supraspinal (including corticospinal) control (Hodapp et al. 2007a, b). Phase-dependent modulation of the *H*-reflex is likely to be generated by presynaptic inhibition (Edamura et al. 1991; Yang and Whelan 1993), within the spinal cord or below the brainstem mechanisms (Hodapp et al. 2007b). Larger locomotor *H*-reflex amplitude in general and abnormal patterns of *H*-reflex and *M*1 and *M*2 stretch reflex modulation are consistent with the expected effects of impaired presynaptic inhibition of these spinal reflex pathways, of both spinal and supraspinal origin.

In addition, it is probable that other inhibitory mechanisms are also impaired, further exacerbating problems due to abnormal stretch reflexes (Taylor et al. 1984; Mailis and Ashby 1990) and changes in spinal motoneurons and interneurons (e.g., persistent inward current) (Hultborn 2003; Gorassini et al. 2004; Hornby et al. 2006; Heckman et al. 2008). Reduced corticospinal activation of the TA that results in weak dorsiflexion and foot drop (Knutsson 1981; Yang et al. 1991; Davey et al. 1999; Barthelemy et al. 2010) would also reduce reciprocal inhibition of the soleus (Boorman et al. 1996) (see also Willerslev-Olsen et al. 2014) even if reciprocal inhibition itself is normal. In participants with SCI, reciprocal inhibition between the plantarflexors (e.g., soleus) and dorsiflexors is often abnormal (Ashby and Wiens 1989; Boorman et al. 1991; Crone et al. 2003; Thompson et al. 2009b; Knikou and Mummidisetty 2011), and would further reduce the suppression of the soleus motoneuron excitability in the stance-swing transition through the late-swing phase. Since TA was active during the swing phase in the (majority of) present participants with incomplete SCI (see Fig. 3b), it is possible that impaired reciprocal inhibition contributed to ineffective suppression of soleus and its stretch reflexes during the swing phase. Ib inhibition (Morita et al. 2006), recurrent inhibition (Shefner et al. 1992), and cutaneous reflexes (Jones and Yang 1994) are also altered after SCI, and these pathways may interact with each other (Bastiaanse et al. 2006; Knikou 2007; Knikou et al. 2007; Nakajima et al. 2014), contributing to spastic locomotor EMG patterns and impaired stretch reflex modulation. Thus, it is probable that multiple inhibitory mechanisms are depressed during walking, resulting in disorganized and ineffective activation of multiple muscles in participants with SCI.

### Functional implications

Normally, the soleus *H*-reflex and all three stretch reflex components are phase-dependently modulated during walking, in accord with soleus EMG activity, which is high in the mid-stance phase and very low (little-to-none) in the swing phase (Capaday and Stein 1986; Sinkjaer et al. 1996a, 1999; Andersen and Sinkjaer 1999). The present results showed that, in individuals with SCI, modulation of soleus EMG activity, *H*-reflex, and spinal *M*1 and *M*2 stretch reflexes was reduced during walking and was abnormal in form. Soleus EMG was unsuppressed during the swing phase but reduced in mid-stance (Fig. 3a). *M*1 and *M*2 were not only unsuppressed during the swing phase, but also often larger in the mid–late-swing phase, which may be explained in part by inadequately hyperactive soleus elevating the reflex excitability. Inversely, enhanced and unsuppressed spinal stretch reflex pathways might contribute to the reduced soleus suppression in the swing phase. These possibilities require further examinations in the future.

In this study, we observed that stretch reflex elicitation in the mid–late-swing phase induces clonus that continues into the stance phase, which would, in turn, interfere with smooth forward motion (Nielsen et al. 1998). Unexpected rapid (stumble-like) stretches used in this study would induce afferent excitation that is faster and more synchronous than that occurring during normal unperturbed walking. However, considering that many individuals with SCI experience toe drag and tripping (i.e., perturbation during the swing phase) often enough during their daily locomotion, it is possible that something similar to what was observed in this study, or of lesser extent, would be experienced by ambulatory individuals with chronic incomplete SCI (it is not unusual to witness individuals with SCI getting stuck in the middle of a step, not being able to propel forward due to toe catching or clonus, during overground walking without stimulation or perturbations, in the laboratory or in the community). Large bursts of clonic activity in the early and early–mid-stance phase enhance generation of posterior breaking force (Turns et al. 2007), reducing forward propulsion. Furthermore, these bursts are often followed by more clonic bursts and/or a much reduced push-off burst in the mid–late stance phase (e.g., Fig. 7), which would reduce propulsive force generation (Turns et al. 2007) and gait speed (Bowden et al. 2006). Thus, individuals with exaggerated spinal stretch reflexes, like those in this study, may encounter these gait obstructions, in response to small extra ankle rotations throughout their daily locomotion.

Together with the present findings, frequent occurrence of clonus in individuals with chronic SCI (e.g., Latash et al. 1989; Rossi et al. 1990; Boyraz et al. 2009, Figs. 7, 8) and presumed contribution of group Ia and II afferents to clonus (Boyraz et al. 2009; Uysal et al. 2009, 2011) urge further investigation of stretch reflexes and its relation to clonus during dynamic motion. To better understand afferent contributions to unperturbed spastic gait, our future plans include stretch reflex examinations using slow small stretches (e.g., Mazzaro et al. 2005, 2007; Willerslev-Olsen et al. 2014) and/or “unloading” perturbations (Sinkjaer et al. 2000).

### Consideration of methodological limitations

Several methodological limitations in the present study would deserve brief discussion. First, in the present study, we did not measure the soleus *M*_max_ across the different phases of walking. Since the soleus *M*_max_ size varies across different ankle angles (Frigon et al. 2007) and across different phases of step cycle (Simonsen and Dyhre-Poulsen 1999; Ferris et al. 2001) with different patterns and extents in different individuals (Simonsen and Dyhre-Poulsen 1999), the locomotor reflex modulation presented here (Fig. 3) may not be completely accurate. Since the locomotor *M*_max_ across the step cycle could remain close to *M*_max_ during natural standing (as shown in Fig. 5 of Kido et al. 2004a), in the present study, all reflex sizes were normalized to the size of soleus *M*_max_ during standing, instead of the locomotor *M*_max_ of a corresponding phase of step cycle. This limitation in normalization is a trade-off to avoiding the potential detrimental effects of making the *M*_max_ measurements on reflex measurements during walking. To find the true *M*_max_ at each bin of the step cycle, a large number of additional stimuli and an even larger number of steps would be required; both of which could result in fatigue and potential alterations of gait in individuals with SCI. While acknowledging this limitation in our data set, we are confident that the reflex and EMG modulation patterns captured in this study reflect existing abnormalities in these individuals with SCI. Clear *M*1, *M*2, and *H*-reflexes seen in EMG (e.g., Figs. 2, 4) in the late-swing phase, for example, are not the products of normalization.

Second, in the present study, the majority of participants with SCI had been on a stable dose of baclofen and/or other anti-spasticity medication (e.g., tizanidine) at the time of experiment. Since those drugs can affect spinal reflex characteristics (Bes et al. 1988; Latash et al. 1989; Corna et al. 1995; Nielsen et al. 2000; Nielsen and Sinkjaer 2000; Maupas et al. 2004; Mirbagheri et al. 2010; Uysal et al. 2011; Mirbagheri 2015), it is probable that the reflex amplitudes and modulation reported here were under influence of such medication. In investigating the locomotor stretch reflex modulation in individuals with chronic incomplete SCI, our aim was to elucidate abnormal reflex activity during their “usual” gait, which had been established over many months to years with or without medication. In individuals who have been on stable doses of medication, temporal removal of medication would disturb not only their physiological normalcy but also their established, not necessarily normal but functional gait. Thus, while acknowledging medication-related limitations in interpretation, we would value the present participants’ data, as they represent (at least partly) a large proportion of individuals with chronic SCI who live their lives with stable anti-spasticity medication and remaining spasticity.

## Conclusion

This study showed for the first time that the short and medium latencies soleus stretch reflexes were abnormally large in the mid–late-swing phase in ambulatory individuals with chronic incomplete SCI. It is possible that multiple spinal and supraspinal pathways that contribute to other CNS populations’ spastic movement disorders cumulatively and/or through interactions among themselves (Dietz and Sinkjaer 2007; Nielsen et al. 2007; Burke et al. 2013; Li and Francisco 2015) could also contribute to the abnormal locomotor reflex modulation observed in the present participants with SCI. The present observations support a possibility that hyperactive spinal stretch reflexes in the mid–late-swing phase can affect the following stance phase. Studies including full measurements of lower extremity kinematics will be necessary in the future, to examine such potential impacts of abnormal stretch reflexes on locomotion in people with chronic incomplete SCI.
